# Supplementary material for: The nucleoid-associated protein IHF acts as a ‘transcriptional domainin’ protein coordinating the bacterial virulence traits with global transcription
Source: Nucleic Acids Res. 2020 Dec 18;49(2):776–90. doi: 10.1093/nar/gkaa1227 (PMC7826290; doi:10.1093/nar/gkaa1227)
Supplement: gkaa1227_Supplemental_Files [file gkaa1227_supplemental_files.zip › Supplementary Data legends.docx]

## Supplementary Data legends

#### Supplementary Tables

For Tables S1, S2, S3, S4, genes are organized by functional categories. Each gene was designated by only one functional category and genes in the same operon were classified into the same category. Column "D" indicates the operon structure in *Dickeya.*

The columns with “O”, “+” and “-” indicate whether a gene is significantly regulated by a specific condition. “O” represents no significant regulation, “+” and “-” represent the significantly up-regulated or down-regulated genes with adjusted pvalue <0.05, respectively.

**Table S1**: Transcriptional response to PGA in WT strain and *ihfA* mutant, at transition to stationary phase

**Table S2**: Transcriptional response to *ihfA* mutation

**Table S3**: Transcriptional response to novobiocin shock in WT strain and *ihfA* mutant

**Table S4**: List of *ihfA*-differentially expressed genes under DNA relaxation only

**Table S5**: List of predicted IHF binding sites at gene promoters.

#### Supplementary Figures

Fig. S1: Density of predicted IHF binding sites **(A)** and A/T bases **(B)** in intergenic regions between convergent, divergent and tandem genes. Error bars represent 95% confidence intervals.

Fig. S2: Transcriptional effect of *fis* **(A)** and *hns* **(B)** mutation, in absence or presence of novobiocin, depending on gene orientation. In contrast to the strong and systematic orientational effect of *ihfA*, only *fis* presents a limited comparable effect in a relaxed chromosome only. Error bars represent 95% confidence intervals.
