# Supplementary figures and images for: The nucleoid-associated protein IHF acts as a ‘transcriptional domainin’ protein coordinating the bacterial virulence traits with global transcription
Source: Nucleic Acids Res. 2020 Dec 18;49(2):776–90. doi: 10.1093/nar/gkaa1227 (PMC7826290; doi:10.1093/nar/gkaa1227)

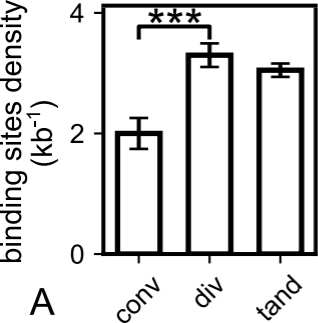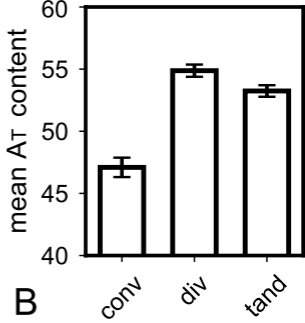

Supplement: gkaa1227_Supplemental_Files [file gkaa1227_supplemental_files.zip › figS1.pdf]

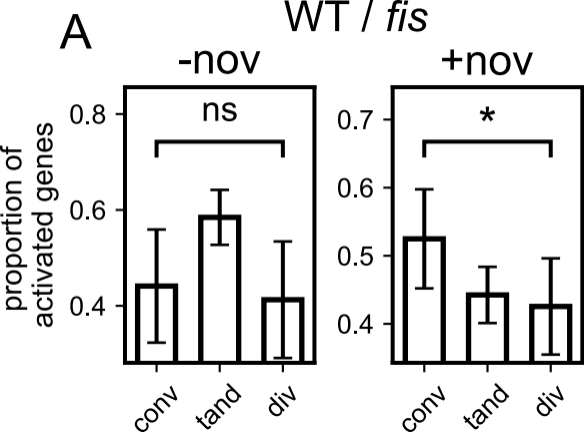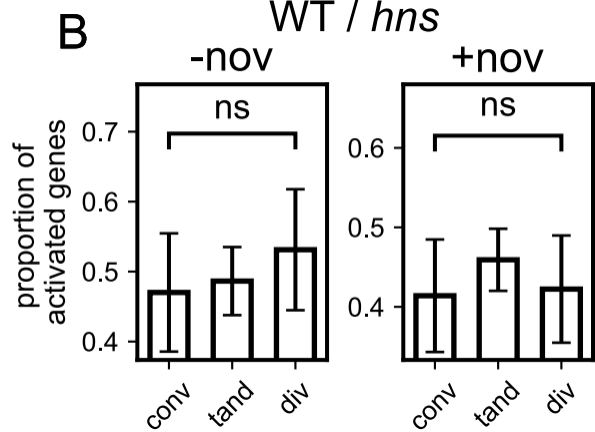

Supplement: gkaa1227_Supplemental_Files [file gkaa1227_supplemental_files.zip › figS2.pdf]
